# Incorporation of biologic factors for the staging of de novo stage IV breast cancer

**DOI:** 10.1038/s41523-020-00186-5

**Published:** 2020-09-07

**Authors:** Zhen-Yu He, Chen-Lu Lian, Jun Wang, Jian Lei, Li Hua, Juan Zhou, San-Gang Wu

**Affiliations:** 1 Department of Radiation Oncology, Sun Yat-sen University Cancer Center, State Key Laboratory of Oncology in South China, Collaborative Innovation Center of Cancer Medicine, 510060 Guangzhou, People’s Republic of China; 2 Department of Radiation Oncology, The First Affiliated Hospital of Xiamen University, 361003 Xiamen, People’s Republic of China; 3 Department of Obstetrics and Gynecology, The First Affiliated Hospital of Xiamen University, 361003 Xiamen, People’s Republic of China

**Keywords:** Prognostic markers, Tumour biomarkers

## Abstract

This study aimed to investigate the prognostic value of biological factors, including histological grade, estrogen receptor (ER), progesterone receptor (PR), and human epidermal growth factor receptor-2 (HER2) status in de novo stage IV breast cancer. Based on eligibility, patient data deposited between 2010 and 2014 were collected from the surveillance, epidemiology, and end results database. The receiver operating characteristics curve, Kaplan–Meier analysis, and Cox proportional hazard analysis were used for analysis. We included 8725 patients with a median 3-year breast cancer-specific survival (BCSS) of 52.6%. Higher histologic grade, HER2-negative, ER-negative, and PR-negative disease were significantly associated with lower BCSS in the multivariate prognostic analysis. A risk score staging system separated patients into four risk groups. The risk score was assigned according to a point system: 1 point for grade 3, 1 point if hormone receptor-negative, and 1 point if HER2-negative. The 3-year BCSS was 76.3%, 64.5%, 48.5%, and 23.7% in patients with 0, 1, 2, and 3 points, respectively, with a median BCSS of 72, 52, 35, and 16 months, respectively (*P* < 0.001). The multivariate prognostic analysis showed that the risk score staging system was an independent prognostic factor associated with BCSS. Patients with a higher risk score had a lower BCSS. Sensitivity analyses replicated similar findings after stratification according to tumor stage, nodal stage, the sites of distant metastasis, and the number of distant metastasis. In conclusion, our risk score staging system shows promise for the prognostic stratification of de novo stage IV breast cancer.

## Introduction

De novo stage IV breast cancer is a rare disease that is considered to be incurable and accounting for ~5% of newly diagnosed breast cancer cases^1^. Earlier, the majority of patients with this type of cancer did not survive for more than 5-years after diagnosis, with a 5-year overall survival (OS) of ~20%^2^. However, with advances in chemotherapy, target therapy, and endocrine therapy, the 5-year OS has now increased to 40% in the modern era of multidisciplinary management^3,4^. The 5-year OS could reach 50% in hormone receptor (HoR)-positive (+) tumors, but the 3-year breast cancer-specific survival (BCSS) and OS for de novo stage IV triple-negative breast cancer are still lower than 20%^5,6^. Further, the median OS for human epidermal growth factor receptor-2 positive (HER2+) tumors in this population has also been reported to reach 60 months after trastuzumab-based therapy^7^, and the prognosis of de novo stage IV disease was found to be better than those with recurrent tumors^8–10^.

Gene expression studies have suggested that the histological grade is more closely related to the molecular composition of breast cancer than the primary tumor size and lymph node status^11,12^. Tumor grade is an important biologic factor that has been incorporated into the most recent breast cancer staging system of the 8th American Joint Committee on Cancer (AJCC)^13^. The 8th AJCC breast cancer staging system has significantly changed from the 7th AJCC anatomical staging system. Biologic factors in breast cancer, including histological grade, HER2, estrogen receptor (ER), and progesterone receptor (PR) status, have been now included in the traditional anatomic primary tumor (T), regional lymph nodes (N), and distant metastasis (M) staging system to create new stages^13^. Several studies have verified that the new staging system is more accurate in predicting prognosis than the 7th AJCC staging system^14–17^. However, the new staging system only includes patients with non-metastatic disease; those in de novo stage IV disease were excluded^13^. In previous studies, including ours, have shown that the HoR+/HER2+ subtype was associated with significantly better BCSS than the HoR+/HER2− and HoR−/HER2+ subtypes in de novo stage IV disease, while those with HoR-/HER2- disease had the worst survival^2,5^. Therefore, tumor biologic factors are significant predictors for both responses to therapy and prognosis in non-metastatic as well as metastatic disease.

The de novo stage IV subgroup is an important enrolled population in clinical trials. Further, there is a significant difference in the survival of this population. Therefore, it is critical to investigate whether the biologic factors based on the 8th AJCC stages could also apply to de novo stage IV disease. In light of this, we explored the prognostic value of biological factors for this disease using a population-based cohort from the surveillance, epidemiology, and end results (SEER) program.

## Results

### Patient characteristics

**Fig. 1:**
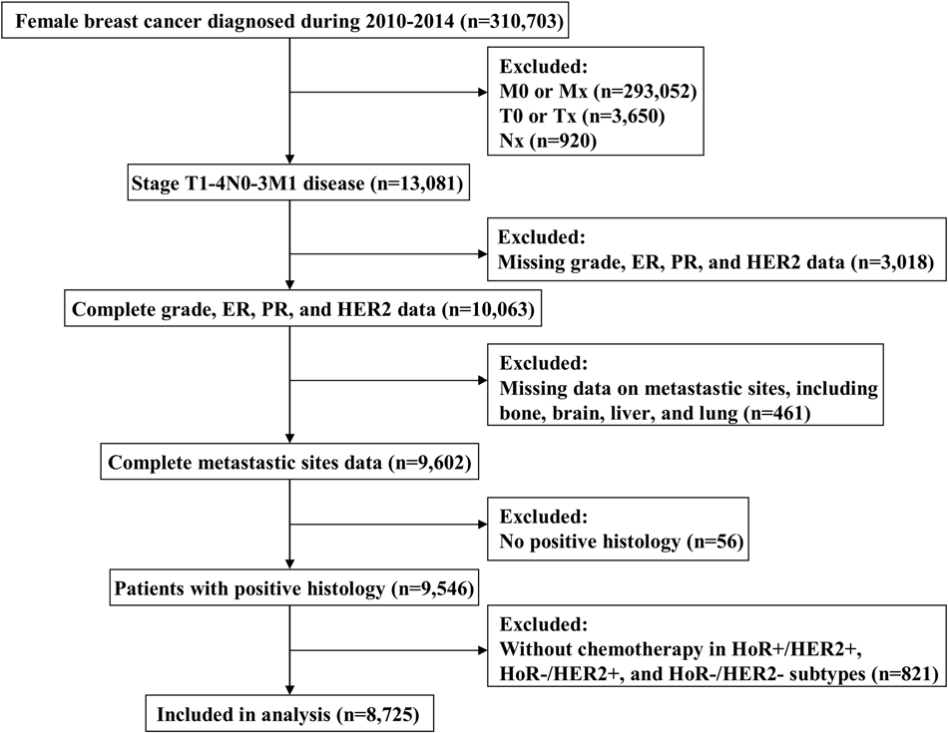
The patient selection flowchart of the study. Flowchart diagram outlining patients included in the analysis and reasons for patient exclusion.

We identified 8725 patients that met the criteria of this study. Figure 1 depicts the patient selection flowchart for this study. The demographic, clinicopathological, treatment, and distant metastasis data of the patients are listed in Table 1. Of the entire cohort, 83.0%, 76.0%, and 51.2% had infiltrating ductal carcinoma, node-positive disease, and T3-4 disease, respectively. In addition, 8.5%, 42.5%, and 49.0% of patients presented with well-differentiated (G1), moderately differentiated (G2), and poorly/undifferentiated (G3) tumors, respectively. Moreover, 5665 (64.9%), 1218 (14.0%), 718 (8.2%), and 1124 (12.9%) patients had the HoR+/HER2−, HoR+/HER2+, HoR−/HER2+, and HoR−/HER2− subtypes, respectively.

**Table 1:** Patients’ demographic, clinicopathological characteristics, treatment, and the patterns of distant metastasis (*N* = 8725).

Variables	*N* (%)
Age (years)	
Mean ± SD (range)	59.7 ± 13.9 (19–103)
Race/ethnicity	
Non-Hispanic White	5647 (64.7)
Non-Hispanic Black	1467 (16.8)
Hispanic (All Races)	941 (10.8)
Other	658 (7.5)
Unknown	12 (0.1)
Grade	
G1	743 (8.5)
G2	3704 (42.5)
G3	4278 (49.0)
Histological subtypes	
Infiltrating ductal carcinoma	7244 (83.0)
Infiltrating lobular carcinoma	875 (10.0)
Other	606 (6.9)
Tumor stage	
T1	1294 (14.8)
T2	2968 (34.0)
T3	1550 (17.8)
T4	2913 (33.4)
Nodal stage	
N0	2098 (24.0)
N1	3914 (44.9)
N2	1183 (13.6)
N3	1530 (17.5)
Hormone receptor status	
ER+ and PR+	5400 (61.9)
ER+ or PR+	1483 (17.0)
ER− and PR−	1842 (21.1)
HER2 status	
Negative	6789 (77.8)
Positive	1936 (22.2)
Surgery	
No	4897 (56.1)
Yes	3793 (43.5)
Unknown	35 (0.4)
Radiotherapy	
No	5327 (61.1)
Yes	3166 (36.3)
Unknown	232 (2.7)
Chemotherapy	
No	3104 (35.6)
Yes	5621 (64.4)
Bone metastasis	
No	3164 (36.3)
Yes	5561 (63.7)
Brain metastasis	
No	8189 (93.9)
Yes	536 (6.1)
Liver metastasis	
No	6636 (76.1)
Yes	2089 (23.9)
Lung metastasis	
No	6174 (70.8)
Yes	2551 (29.2)
Other metastatic sites	
No	7497 (85.9)
Yes	1228 (14.1)
Number of metastatic sites (*n* = 7497)^a^	
1	4963 (66.2)
2	1908 (25.5)
3	546 (7.3)
4	80 (1.1)

*ER* estrogen receptor, *G1* well-differentiated, *G2* moderately differentiated, *G3* poorly/undifferentiated, *HER2* human epidermal growth factor receptor-2, *N* nodal, *PR* progesterone receptor, *SD* standard deviation, *T* tumor.

^a^Indicates four metastatic sites, including bone, brain, liver, and lung.

A total of 5561 (63.7%), 2551 (29.2%), 2089 (23.9%), and 536 (6.1%) patients presented with bone, lung, liver, and brain metastasis, respectively. In patients for whom information about these four metastatic sites were available (*n* = 7497), 4963 (66.2%), 1908 (25.5%), 546 (7.3%), and 80 (1.1%) had one, two, three, and four metastatic sites, respectively.

### Survival and prognosis

Within a median follow-up of 29 months (range, 0–83 months), there were 5326 deaths observed, out of which 4653 were related to breast cancer. The 3-year BCSS was 52.6%, and the median BCSS was 39 months.

Multivariate analysis showed that higher histologic grade, HER2-negative, single HoR-positive (ER-positive or PR-positive), and double HoR-negative (ER-negative and PR- negative) status were significantly associated with lower BCSS (Table 2). Moreover, age, race/ethnicity, histology, surgery, chemotherapy, bone metastasis, lung metastasis, liver metastasis, and brain metastasis were also identified as independent prognostic factors correlated with BCSS. However, the BCSS was comparable among patients with stage T1 and T2 disease, and BCSS was also comparable among patients with stage N0, N1, N2, and N3 disease.

**Table 2:** Multivariate prognostic analysis, including histologic grade, ER, PR, and HER2 status.

Variables	HR	95% CI	*P*
Age (years) (continuous variable)	1.015	1.013–1.018	<0.001
Race/ethnicity			
Non-Hispanic White	1		
Non-Hispanic Black	1.331	1.233–1.438	<0.001
Hispanic (All Races)	1.023	0.928–1.128	0.646
Other	0.988	0.879–1.110	0.841
Unknown	0.262	0.065–1.049	0.058
Grade			
G1	1		
G2	1.379	1.217–1.556	<0.001
G3	1.934	1.704–2.195	<0.001
Histological subtypes			
Infiltrating ductal carcinoma	1		
Infiltrating lobular carcinoma	1.213	1.096–1.342	<0.001
Other	1.181	1.060–1.317	0.003
Tumor stage			
T1	1		
T2	1.007	0.915–1.108	0.891
T3	1.114	1.001–1.239	0.048
T4	1.289	1.170–1.420	<0.001
Nodal stage			
N0	1		
N1	0.965	0.894–1.040	0.350
N2	1.022	0.922–1.133	0.678
N3	1.084	0.987–1.191	0.090
Hormone receptor status			
ER+ and PR+	1		
ER+ or PR+	1.772	1.639–1.916	<0.001
ER− and PR−	2.261	2.078–2.459	<0.001
HER2 status			
Negative	1		
Positive	0.414	0.381–0.450	<0.001
Surgery			
No	1		
Yes	0.601	0.563–0.641	<0.001
Unknown	1.146	0.737–1.781	0.546
Radiotherapy			
No	1		
Yes	0.986	0.927–1.051	0.662
Unknown	0.807	0.656–0.992	0.042
Chemotherapy			
No	1		
Yes	0.917	0.851–0.988	0.023
Bone metastasis			
No	1		
Yes	1.266	1.187–1.350	<0.001
Brain metastasis			
No	1		
Yes	2.141	1.928–2.377	<0.001
Liver metastasis			
No	1		
Yes	1.771	1.655–1.895	<0.001
Lung metastasis			
No	1		
Yes	1.223	1.147–1.303	<0.001
Other metastatic sites			
No	1		
Yes	1.021	0.907–1.149	0.730

*CI* confidence interval, *ER* estrogen receptor, *G1* well-differentiated, *G2* moderately differentiated, *G3* poorly/undifferentiated, *HER2* human epidermal growth factor receptor-2, *N* nodal, *HR* hazard ratio, *PR* progesterone receptor, *T* tumor.

According to the status of the included biologic factors, such as tumor grade, HER2 status, ER status, and PR status, a total of 24 subgroups were reclassified (Table 3). Of these patients, no patients were assigned to the G1/HER2+/ER−/PR+ subgroup, and the top five common subgroups were G2/HER2−/ER+/PR+ (28.6%), G3/HER2−/ER+/PR+ (17.4%), G3/HER2−/ER−/PR− (10.7%), G1/HER2−/ER+/PR+ (7.1%), and G3/HER2+/ER−/PR− (6.1%). According to Kaplan–Meier analysis, significantly longer median BCSS was found in the G1/HER2+/ER+/PR+ and G1/HER2+/ER+/PR− subgroups, and the median BCSS was not reached in these subgroups. The worst median BCSS was observed in three subgroups: G1/HER2−/ER−/PR+, G2/HER2−/ER−/PR+, and G3/HER2−/ER−/PR+, which had a median BCSS of 4, 10, and 11 months, respectively.

**Table 3:** The median BCSS in 24 subgroups according to the histological grade, ER, PR, and HER2 status.

Tumor grade	HER2 status	ER status	PR status	*N* (%)	Median BCSS (months)	Risk score (MDACC)	Risk score (SEER)
G1	HER2+	ER+	PR+	18 (0.2)	NA	0	0
G1	HER2+	ER+	PR−	5 (0.1)	NA	0	1
G1	HER2+	ER−	PR+	NA	NA	1	1
G1	HER2+	ER−	PR−	4 (0.1)	72	1	1
G1	HER2−	ER+	PR+	617 (7.1)	63	1	1
G1	HER2−	ER+	PR−	88 (1.0)	35	1	2
G1	HER2−	ER−	PR+	2 (0.1)	4	2	2
G1	HER2−	ER−	PR−	9 (0.1)	18	2	2
G2	HER2+	ER+	PR+	290 (3.3)	72	0	0
G2	HER2+	ER+	PR−	133 (1.5)	47	0	1
G2	HER2+	ER−	PR+	16 (0.2)	39	1	1
G2	HER2+	ER−	PR−	186 (2.1)	49	1	1
G2	HER2−	ER+	PR+	2493 (28.6)	50	1	1
G2	HER2−	ER+	PR−	393 (4.5)	27	1	2
G2	HER2−	ER−	PR+	13 (0.1)	10	2	2
G2	HER2−	ER−	PR−	180 (2.1)	17	2	2
G3	HER2+	ER+	PR+	461 (5.3)	65	1	1
G3	HER2+	ER+	PR−	268 (3.1)	44	1	2
G3	HER2+	ER−	PR+	27 (0.3)	52	2	2
G3	HER2+	ER−	PR−	528 (6.1)	49	2	2
G3	HER2−	ER+	PR+	1521 (17.4)	36	2	2
G3	HER2−	ER+	PR−	453 (5.2)	20	2	3
G3	HER2−	ER−	PR+	88 (1.0)	11	3	3
G3	HER2−	ER−	PR−	932 (10.7)	15	3	3

*BCSS* breast cancer-specific survival, *ER* estrogen receptor, *G1* well-differentiated, *G2* moderately differentiated, *G3* poorly/undifferentiated, *HER2* human epidermal growth factor receptor-2, *MDACC* MD Anderson Cancer Center, *NA* none available, *PR* progesterone receptor, *SEER* surveillance, epidemiology, and end results.

### Risk score staging system

**Fig. 2:**
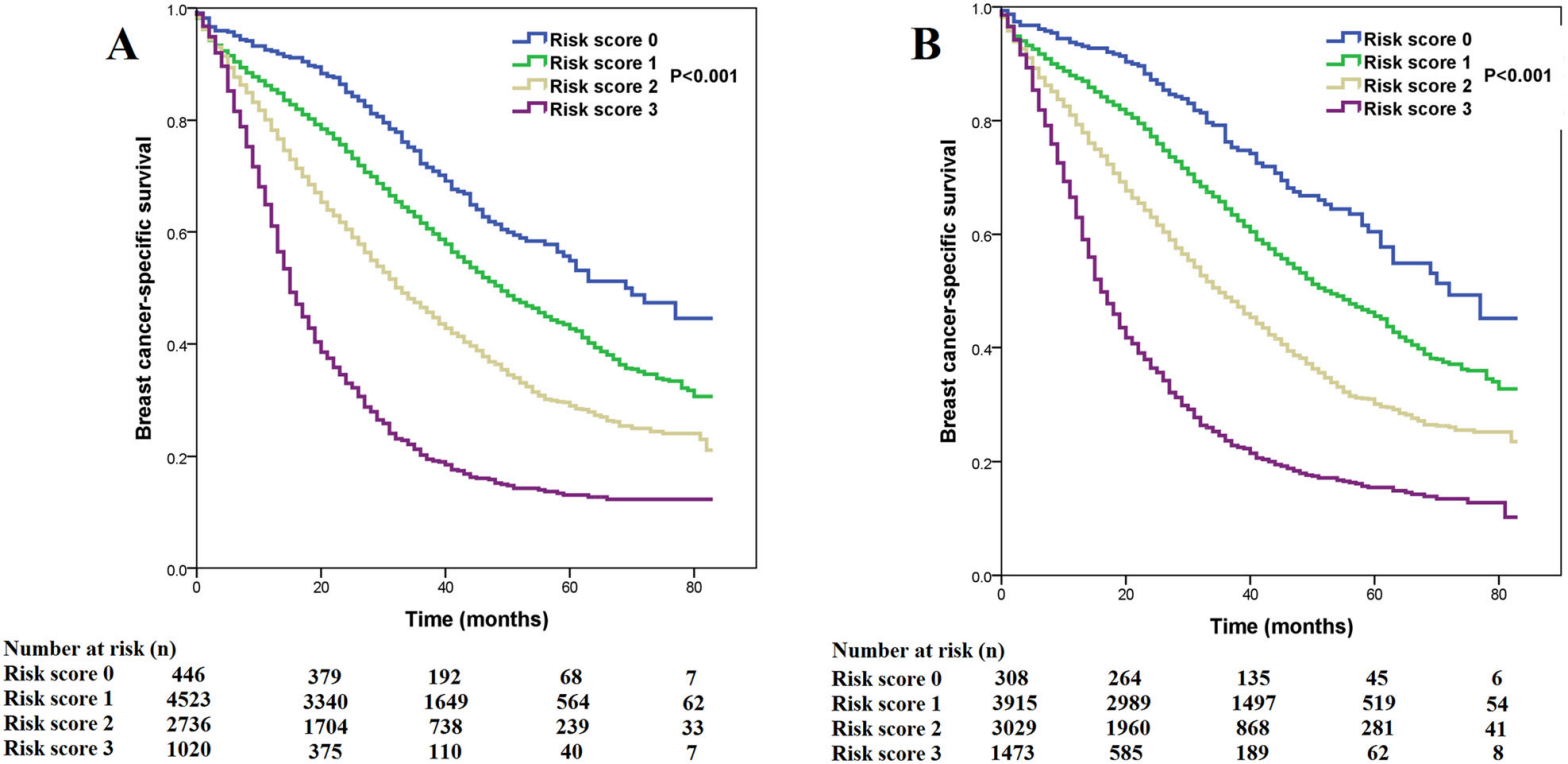
Comparison of breast cancer-specific survival by risk score. **a** MD Anderson Cancer Center risk score staging system; **b** SEER risk score staging system.

**Fig. 3:**
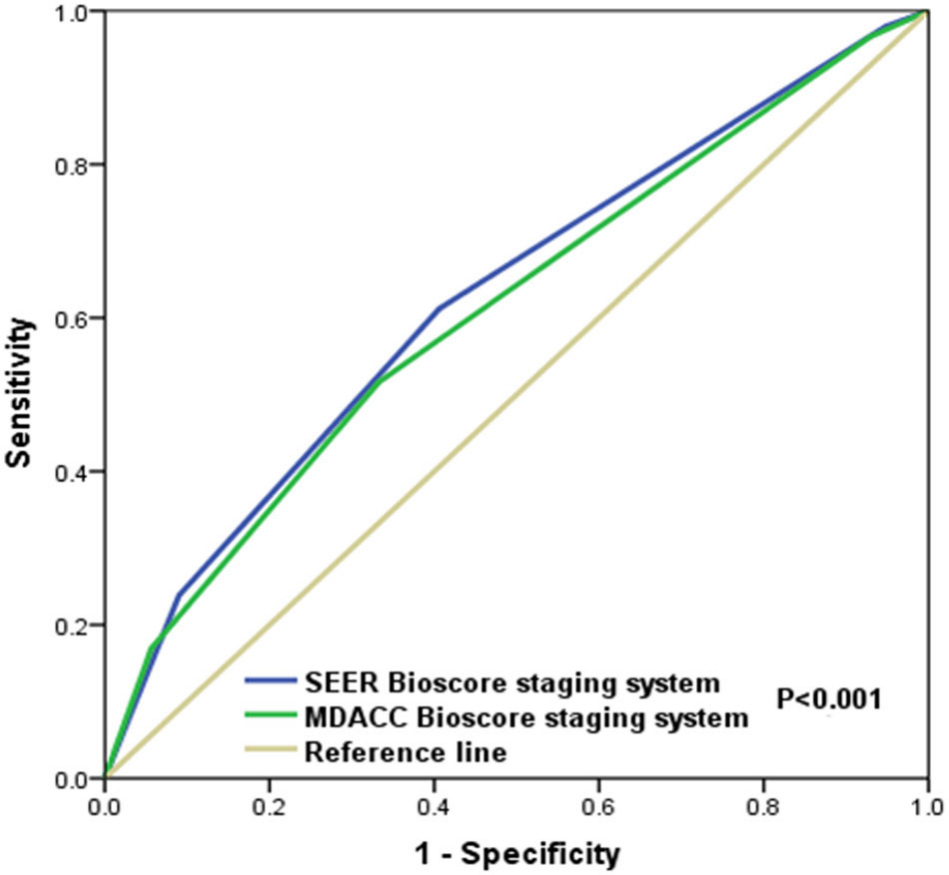
Receiver operating characteristics analyses for prediction of breast cancer-specific survival with the two risk score staging systems. The SEER risk score staging system had a better predictive performance for breast cancer-specific survival compared to the MD Anderson Cancer Center risk score staging system.

A previous study by Chavez–MacGregor et al. from University of Texas MD Anderson Cancer Center (MDACC) developed a risk score staging system based on a risk score of 0–3 assigned according to the histological grade (0 point for G1-2, 1 point for G3), ER status (0 point if ER-positive, 1 point if ER-negative), and HER2 status (0 point if HER2-positive, 1 point if HER2-negative) to stratify the prognosis of patients (Table 3)^18^. With the MDACC risk score staging system, the 3-year BCSS was 72.3%, 61.6%, 46.5%, and 20.2% in patients with 0, 1, 2, and 3 points, respectively, with a median BCSS of 69, 49, 33, and 15 months, respectively (Fig. 2a). In our study, the worst median BCSS was observed in the subgroups: G1-3/HER2−/ER−/PR+, which indicated that patients with single HoR-positive disease had inferior BCSS compared to those with double HoR-positive disease. Although there was a significant difference in BCSS between single HoR-positive and double HoR-negative diseases using the Kaplan–Meier analysis (*P* = 0.012), the survival curves were overlapped. Therefore, we integrated single HoR-positive and double HoR-negative cohorts into an aggressive subgroup. We developed another risk score staging system (SEER database) based on histological grade (0 point for G1-2, 1 point for G3), HER2 status (0 point if HER2-positive, 1 point if HER2-negative), HoR status (0 point if double HoR-positive, 1 point if single HoR-positive or double HoR-negative). The score ranges from 0 to 3, and BCSS could also be predicted according to this risk score. Within the SEER risk score staging system, the 3-year BCSS was 76.3%, 64.5%, 48.5%, and 23.7% in patients with 0, 1, 2, and 3 points, respectively, with a median BCSS of 72, 52, 35, and 16 months, respectively (Fig. 2b). The SEER risk score staging system was examined against the MDACC risk score staging system using the receiver operating characteristics (ROC) curve. The area under the curve (AUC) under the ROC curve of SEER risk score staging system (AUC = 0.628, 95%CI 0.618–0.638) was significantly higher than that of MDACC risk score staging system (AUC = 0.611, 95%CI 0.601–0.622) (*P* < 0.0001) (Fig. 3). The results indicated that the SEER risk score staging system had a better predictive performance for BCSS compared to the MDACC risk score staging system.

### Prognostic value of the risk score staging system

**Fig. 4:**
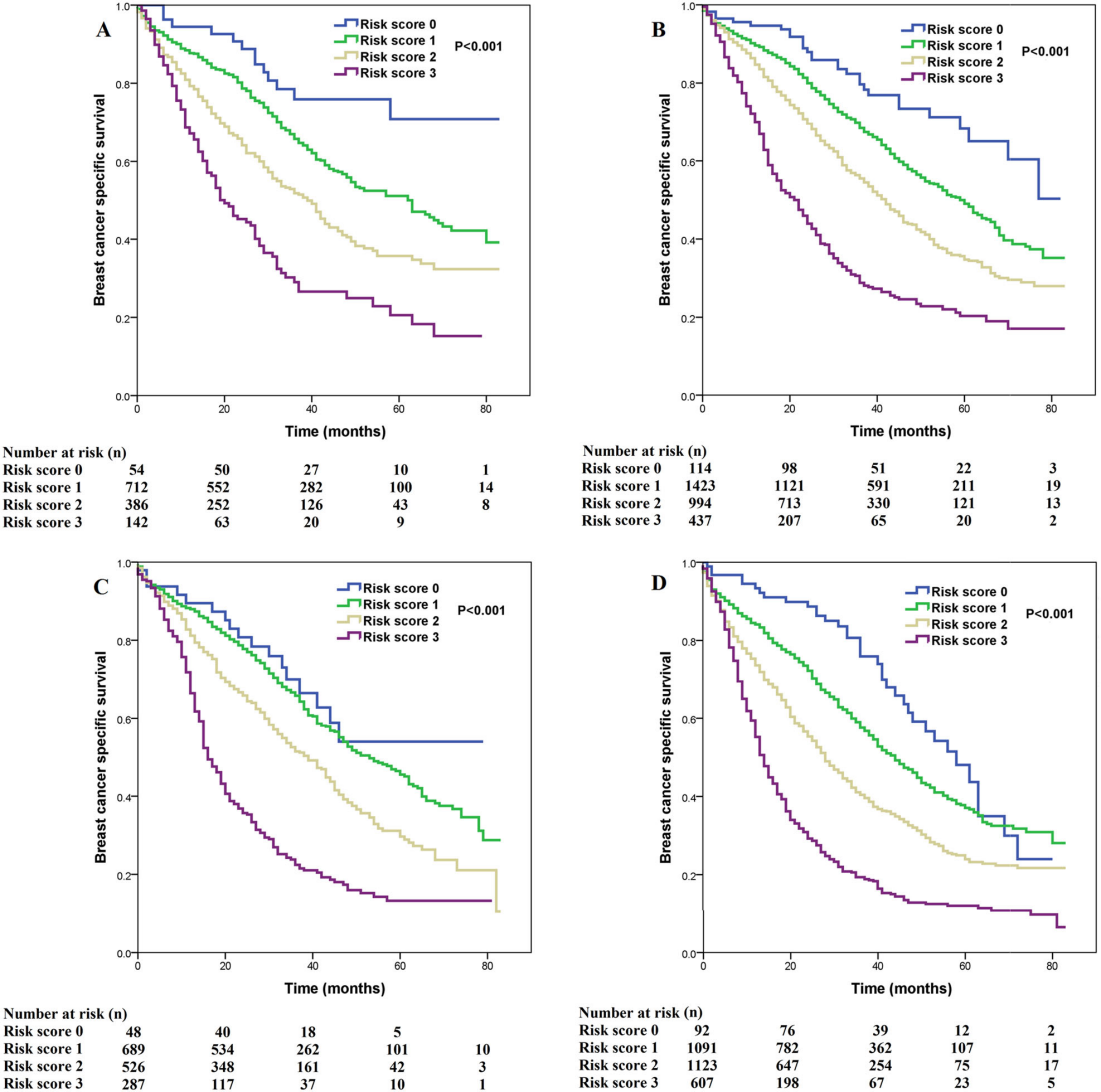
Comparison of breast cancer-specific survival by risk score for T1-4 patients using the SEER risk score staging system. **a** T1; **b** T2; **c** T3; **d** T4.

**Fig. 5:**
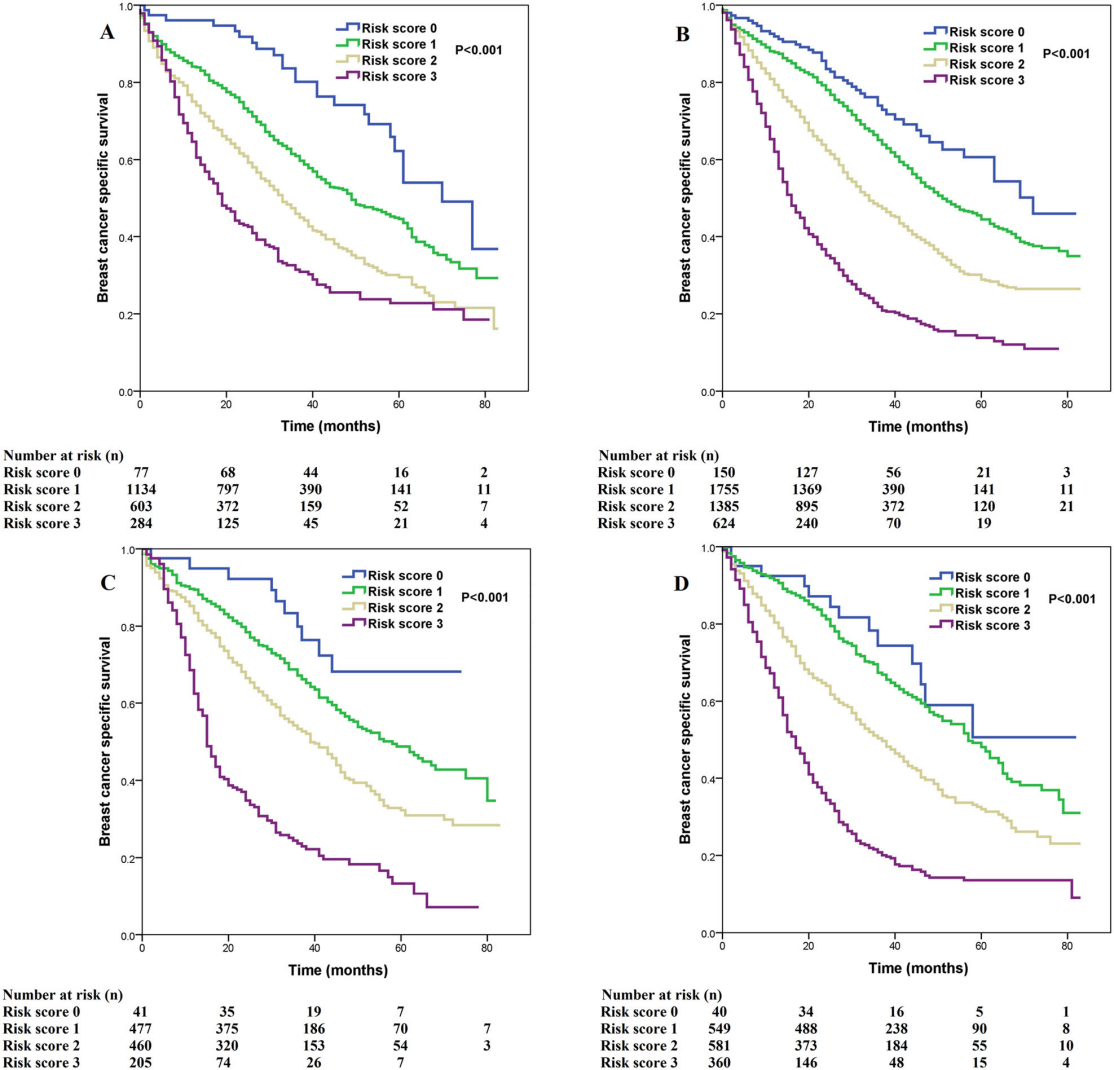
Comparison of breast cancer-specific survival by risk score for N0-3 patients using the SEER risk score staging system. **a** N0; **b** N1; **c** N2; **d** N3.

**Fig. 6:**
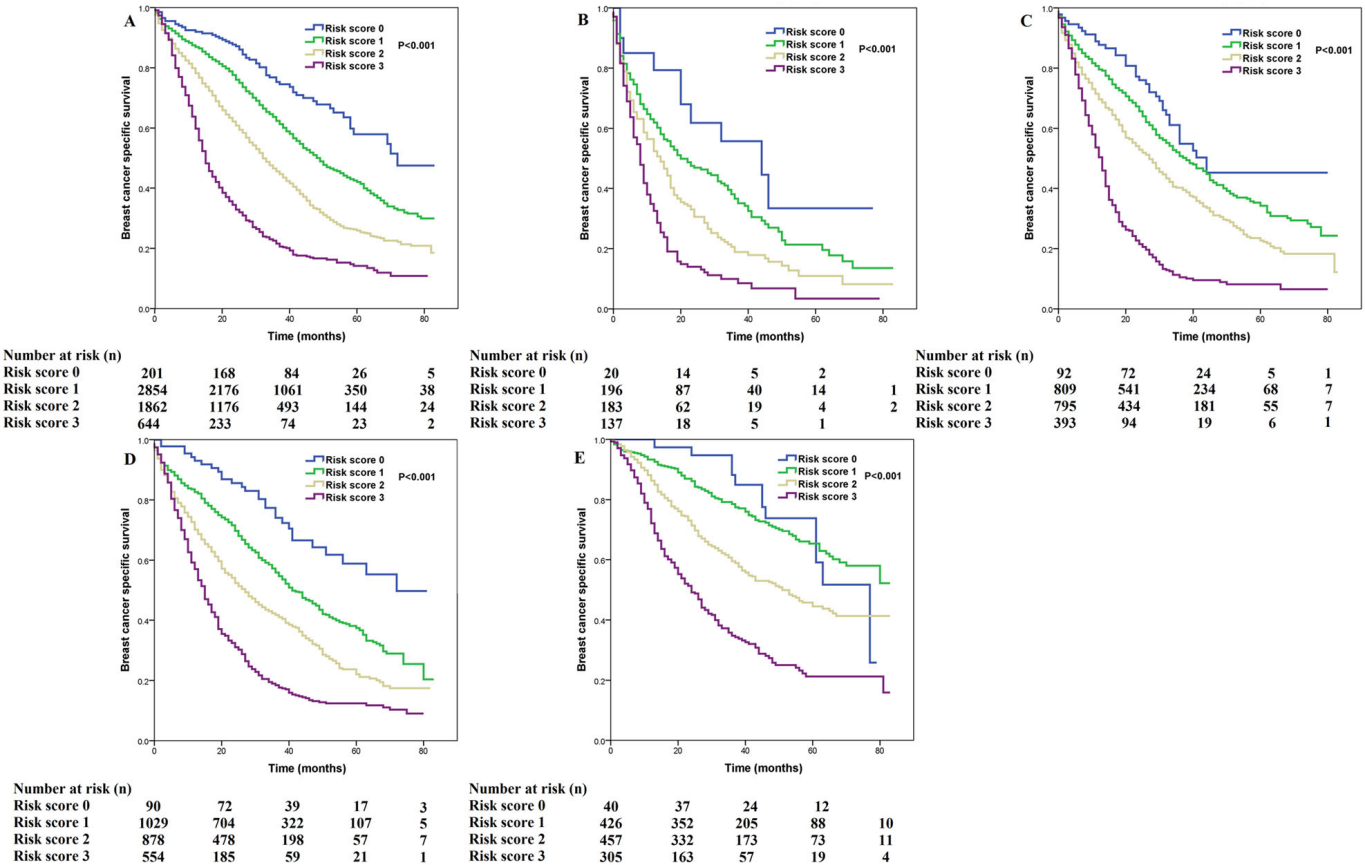
Comparison of breast cancer-specific survival by risk score in different metastatic sites using the SEER risk score staging system. **a** bone; **b** brain; **c** liver; **d** lung; **e** other.

**Fig. 7:**
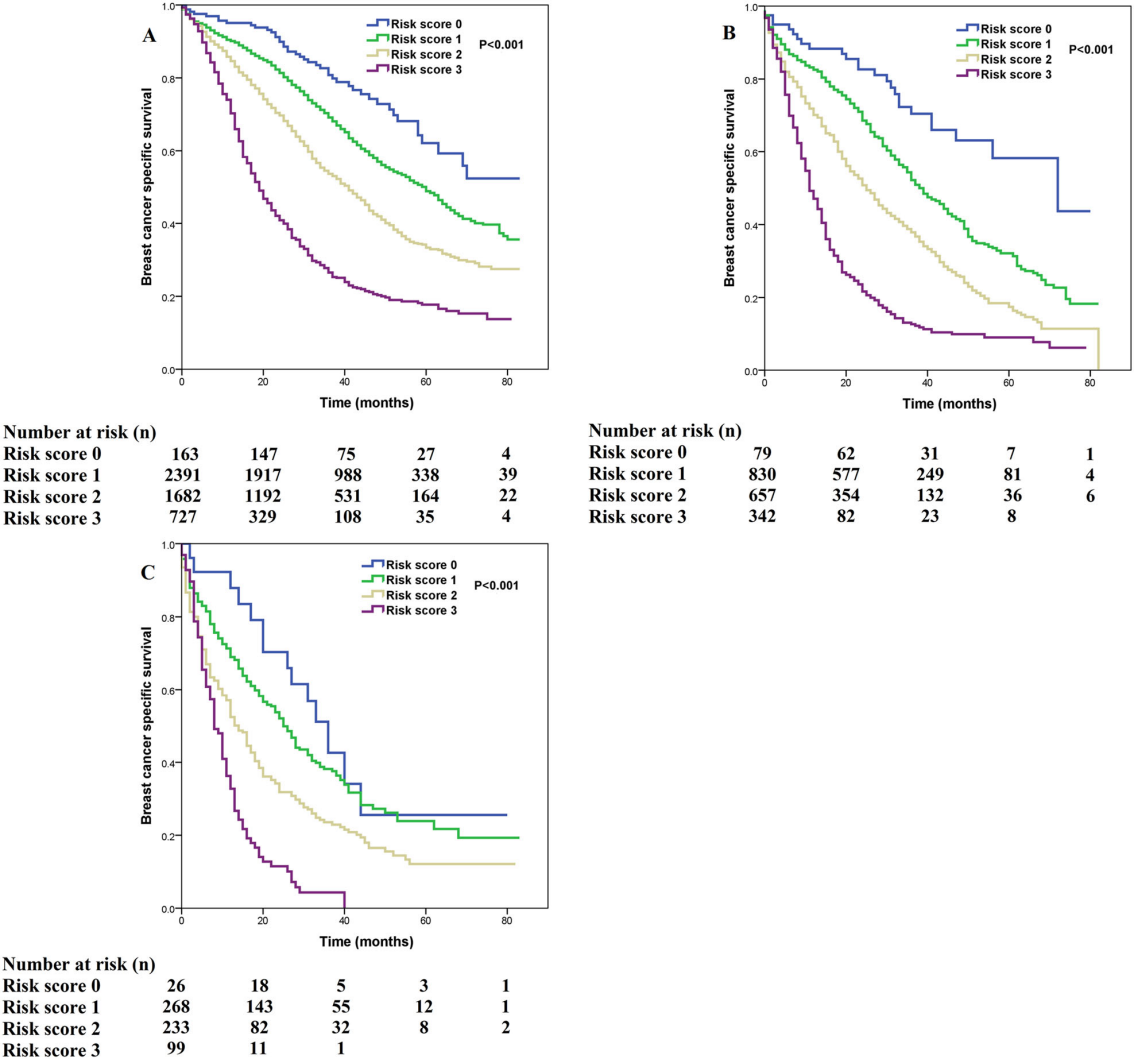
Comparison of breast cancer-specific survival by risk score in different number of metastatic sites using the SEER risk score staging system. **a** one site; **b** two sites; **c** three-four sites.

We used multivariate prognostic analysis to assess the prognostic effect of the SEER risk score staging system based on BCSS (Table 4). After adjustment for age, race/ethnicity, histology, T stage, N stage, surgery, chemotherapy, radiotherapy, and the sites of distant metastasis, the SEER risk score staging system was found to be an independent prognostic factor associated with BCSS. Patients with a higher risk score had a lower BCSS. When risk score 0 was used as a reference, patients with risk score 1 was associated with significantly lower BCSS than those with risk score 0 (hazard ratio [HR] = 1.473, 95% confidence interval [CI] = 1.195–1.816, *P* < 0.001), patients with risk score 2 had a significantly lower BCSS than those with risk score 0 (HR = 2.437, 95% CI = 1.979–3.001, *P* < 0.001), patients with risk score 3 had a significantly lower BCSS compared to those with risk score 0 (HR = 5.092, 95% CI = 4.121–6.291, *P* < 0.001). Patients with risk score 3 was associated with significantly lower BCSS compared to those with risk score 1 (HR = 3.456, 95% CI = 3.182–3.754, *P* < 0.001) and risk score 2 (HR = 2.647, 95% CI = 2.459–2.848, *P* < 0.001). Sensitivity analyses replicated similar findings after stratification according to the T stage (Fig. 4a–d), N stage (Fig. 5a–d), the sites of distant metastasis (Fig. 6a–e), and the number of distant metastasis (Fig. 7a–c) (Table 5).

**Table 4:** Multivariate prognostic analysis, including risk score staging system.

Variables	HR	95% CI	*P*
Age (years) (continuous variable)	1.015	1.013–1.018	<0.001
Race/ethnicity			
Non-Hispanic White	1		
Non-Hispanic Black	1.334	1.235–1.441	<0.001
Hispanic (All Races)	1.010	0.916–1.114	0.844
Other	0.982	0.874–1.103	0.757
Unknown	0.264	0.066–1.059	0.060
Histological subtypes			
Infiltrating ductal carcinoma	1		
Infiltrating lobular carcinoma	1.251	1.132–1.382	<0.001
Other	1.175	1.054–1.309	0.004
Tumor stage			
T1	1		
T2	1.010	0.918–1.110	0.843
T3	1.129	1.016–1.254	0.024
T4	1.292	1.176–1.420	<0.001
Nodal stage			
N0	1		
N1	0.955	0.886–1.030	0.236
N2	1.001	0.903–1.109	0.989
N3	1.055	0.960–1.158	0.267
Risk stratification (SEER)			
Risk score 0	1		
Risk score 1	1.473	1.195–1.816	<0.001
Risk score 2	2.437	1.979–3.001	<0.001
Risk score 3	5.092	4.121–6.291	<0.001
Surgery			
No	1		
Yes	0.608	0.570–0.648	<0.001
Unknown	1.112	0.715–1.728	0.637
Radiotherapy			
No	1		
Yes	0.992	0.930–1.058	0.804
Unknown	0.796	0.648–0.979	0.031
Chemotherapy			
No	1		
Yes	0.876	0.817–0.938	<0.001
Bone metastasis			
No	1		
Yes	1.262	1.183–1.345	<0.001
Brain metastasis			
No	1		
Yes	2.128	1.910–2.371	<0.001
Liver metastasis			
No	1		
Yes	1.703	1.593–1.821	<0.001
Lung metastasis			
No	1		
Yes	1.216	1.141–1.296	<0.001
Other metastatic sites			
No	1		
Yes	1.029	0.914–1.158	0.639

*CI* confidence interval, *HR* hazard ratio, *N* nodal, *T* tumor, *SEER* surveillance, epidemiology, and end results.

**Table 5:** Adjusted hazard ratios and the median BCSS in the risk score staging system in different subgroups.

Stage	Risk score	Median BCSS (months)	*P*	HR	95% CI	*P*	Patterns of distant metastasis	Risk score	Median BCSS (months)	*P*	HR	95% CI	*P*
T1	0	NA	<0.001	1			Bone	0	72	<0.001	1		
	1	62		1.847	1.059–3.223	0.031		1	48		1.606	1.238–2.084	<0.001
	2	39		3.177	1.814–5.566	<0.001		2	32		2.636	2.032–3.419	<0.001
	3	19		5.664	3.163–10.145	<0.001		3	15		5.048	3.859–6.602	<0.001
T2	0	NA	<0.001	1			Brain	0	44	<0.001	1		
	1	59		1.601	1.101–2.327	0.014		1	20		1.866	0.979–3.559	0.058
	2	42		2.571	1.767–3.740	<0.001		2	14		2.970	1.558–5.661	0.001
	3	21		5.666	3.859–8.321	<0.001		3	8		4.996	2.588–9.645	<0.001
T3	0	NA	<0.001	1			Liver	0	44	<0.001	1		
	1	53		1.065	0.648–1.749	0.804		1	38		1.256	0.898–1.756	0.183
	2	39		1.685	1.029–2.757	0.038		2	27		1.987	1.426–2.769	<0.001
	3	16		3.856	2.341–6.352	<0.001		3	13		4.275	3.040–6.010	<0.001
T4	0	58	<0.001	1			Lung	0	72	<0.001	1		
	1	44		1.383	0.982–1.948	0.063		1	41		1.893	1.301–2.755	0.001
	2	28		2.374	1.692–3.329	<0.001		2	27		3.144	2.162–4.573	<0.001
	3	14		4.780	3.394–6.733	<0.001		3	15		5.933	4.059–8.670	<0.001
N0	0	70	<0.001	1			Other sites	0	77	<0.001	1		
	1	49		1.853	1.228–2.796	0.003		1	NA		0.866	0.474–1.582	0.640
	2	33		2.818	1.861–4.267	<0.001		2	51		1.644	0.915–2.954	0.096
	3	19		4.698	3.059–7.216	<0.001		3	24		3.804	2.119–6.830	<0.001
N1	0	72	<0.001	1			One site^a^	0	NA	<0.001	1		
	1	51		1.258	0.936–1.692	0.128		1	59		1.501	1.104–2.040	0.010
	2	34		2.165	1.613–2.904	<0.001		2	41		2.414	1.778–3.277	<0.001
	3	16		4.694	3.479–6.334	<0.001		3	19		5.182	3.800–7.066	<0.001
N2	0	NA	<0.001	1			Two sites^a^	0	72	<0.001	1		
	1	58		1.439	0.758–2.731	0.266		1	38		1.901	1.270–2.846	0.002
	2	39		2.348	1.224–4.432	0.008		2	25		3.007	2.008–4.502	<0.001
	3	15		5.602	2.935–10.692	<0.001		3	11		5.601	3.714–8.448	<0.001
N3	0	NA	<0.001	1			Three-four sites^a^	0	36	<0.001	1		
	1	57		1.683	0.955–2.964	0.072		1	25		1.217	0.698–2.121	0.488
	2	37		2.892	1.649–5.073	<0.001		2	14		2.139	1.235–3.707	0.007
	3	17		6.581	3.736–11.596	<0.001		3	8		3.632	2.041–6.463	<0.001

*BCSS* breast cancer-specific survival, *CI* confidence interval, *HR* hazard ratio, *N* nodal, *NA* none available, *T* tumor.

^a^Indicates four metastatic sites, including bone, brain, liver, and lung.

## Discussion

A primary limitation of the AJCC 8th stages is that it is limited to patients with non-metastatic breast cancer. It is critical to investigate whether the biologic factors based on the 8th AJCC stages could also be applied to breast cancer with de novo stage IV disease. In the present study, we used a population-based cohort from the SEER program to investigate the prognostic effect of biologic factors in de novo stage IV breast cancer. The current study indicated that the risk score staging system developed by the histological grade, HER2 status, ER status, and PR status might provide a better risk stratification for this population.

The findings in our study may have potential clinical implications in the current era of personalized therapy for de novo stage IV breast cancer. First, it provides a concise summary of the de novo stage IV breast cancer, which allows for efficient communication among clinicians and researchers. In addition, it also provides a framework for relaying prognostic stratification based on the sum of the tumor and biologic factors. According to this prognostic framework, the risk score staging system can be applied to determine the optimal treatment approach for individual patients. Moreover, it can more thoroughly and accurately assess the impact of the novel or changing treatment approach for this population. Finally, the risk score staging system can frequently be used to define subgroups for inclusion in clinical trials.

The present analysis reveals the heterogeneity in the prognosis of the de novo stage IV breast cancer, and therefore, overcomes a significant limitation of the latest AJCC staging system, which does not include de novo stage IV disease. Although several studies have incorporated the biological factors into the substages of this population, only the histologic grade, ER, and HER2 were included in the scoring system for stratification, and the PR status was excluded^18,19^. Another limitation of the previous studies was that the survival curves between risk score 0 and risk score 1 overlapped^18,19^. In this study, a large cohort was used, and the BCSS curves could be clearly distinguished. Additionally, the risk score staging system developed in our study using the data from the SEER program (including grade, HER2, ER, and PR status) had a better predictive performance for BCSS than the MDACC risk score staging system (including grade, HER2, and ER status)^18^. Therefore, in order to better predict the prognosis and guide treatment decisions, these substages based on the risk score staging system should be introduced in the advanced setting similar to patients with non-metastatic disease. Additionally, the risk stratification based on the risk score staging system will undoubtedly serve as critical roles in patient care and research for this population.

Triple-negative breast cancer had the worst outcomes in de novo stage IV disease^2,5^. In our study, we found that the median BCSS was less than 20 months in triple-negative breast cancer patients regardless of the histologic grades. However, it should be noted that in HER2-negative tumors, single HoR-positive tumors (ER+/PR− or ER−/PR+ subtypes) had lower BCSS than those of the double HoR-positive tumors, and had comparable BCSS to those of the double HoR-negative tumors. Our findings were similar to the findings of Bae et al., which indicated that a significant difference in prognosis between single HoR-positive tumors and double HoR-positive tumors, was only observed in HER2-negative tumors, and not in HER2-positive tumors^20^. Several studies also confirmed that single HoR-positive tumors showed worse prognosis than double HoR-positive tumors in the HER2-negative group^21–24^. No significant effect of single HoR-positive tumors in the prognostic assessment of HER2-positive tumors may be related to the results of trastuzumab treatment. In the 8th AJCC staging system, prognostic stage groups were determined in the breast cancer patients that mostly underwent appropriate multidisciplinary treatment, including chemotherapy, anti-HER2 therapy, and endocrine therapy^13^. In our study, all patients with HoR+/HER2+, HoR−/HER2+, and HoR−/HER2− subtypes were received chemotherapy, and approximately half of the HoR+/HER2− patients received chemotherapy. However, we did not have data regarding anti-HER2 therapy and endocrine therapy in this study. In our study, the effect of biological factors on the survival trends in de novo stage IV breast cancer was similar to the results from non-metastatic breast cancer^14–17^. Therefore, we could assume that the majority of patients in our SEER-based study also received appropriate multidisciplinary treatment according to the status of biologic factors.

According to the 8th AJCC pathological staging system, T2N0M0, G2/HER2−/ER+/PR+patients are classified as stage IA, and G2/HER2−/ER+PR−, G2/HER2−/ER−/PR+, G2/HER2−/ER−/PR− patients are classified as stage IIB^13^. Furthermore, consistent with our findings, the survival of HER2-negative and single HoR-positive tumors was comparable to that of double HoR-negative tumors according to the new AJCC pathological staging system. The aggressive behavior of single HoR-positive tumors indicated that the single HoR-positive tumors had distinct clinical and biological features. Therefore, in this study, we integrated single HoR-positive and double HoR-negative tumors into an aggressive subgroup. A recent study showed that the HER2−/ER+/PR− subtype exhibited more *ZNF703* and *RPS6KB1* amplification events than HER2−/ER+/PR+ tumors^25^, which could promote cell proliferation, increase the stem cell population, chemotherapy resistance, tamoxifen resistance, and radiotherapy resistance^25–30^. Therefore, further exploration of treatment strategies for single HoR-positive tumors are needed in the future to improve patient survival.

The 8th AJCC staging system incorporates the T stage, N stage, histologic grade, ER, PR, and HER2 status in the determination of the novel stages^13^, but we did not include the T stage and N stage in this study due to the controversial effect of T and N stage on BCSS in patients with *de novo* stage IV breast cancer. Additionally, Li et al. reported that there was no difference in survival between node-negative and node-positive patients^10^. Moreover, the current AJCC staging is mainly divided into clinical staging (all patients for clinical classification and staging) and pathological staging (for patients in whom surgery is the initial treatment), but the role of surgery in de novo stage IV disease remains controversial^3,4,31–33^. Therefore, the significance of integrating T and N stages into the risk score staging system needs to be further explored in the future for this population.

An important caveat should be noted that the patients enrolled in the determination of AJCC 8th stages were treated with multimodal therapy according to the status of biologic factors. However, standard testing of biologic markers for evidence-based treatment might not be accessible to the majority of patients around the globe, especially those in low- and middle-income countries^34^. Thus, the applicability of the risk score staging system to global patients may be compromised.

The role of local management in patients with de novo stage IV breast cancer remains controversial. In our study, we found that local surgery was associated with better BCSS for this population. However, conflict results were reported in the American Society of Clinical Oncology 2020 data. A retrospective study using the data from the National Cancer Database showed that primary tumor resection was associated with better overall survival in breast cancer patients with de novo stage IV disease^35^. Another randomized trial from E2108 indicated that additional locoregional treatment to optimal systemic therapy did not improve progression-free survival or overall survival compared to those in optimal systemic therapy alone arm^36^. According to our findings, it is worth carrying out further study to investigate the role of local management in de novo stage IV breast cancer after stratification by the risk score staging system.

Several limitations of the present analysis should be emphasized. First, the SEER database lacks sufficient details of the chemotherapy regimen, endocrine therapy, and anti-HER2 therapy. Second, comorbidity and performance status are also not recorded in the SEER database. Third, our study used BCSS in order to neutralize any confounding effects resulting from non-breast cancer-related death. In addition, the SEER program lacks a central pathology review for the biologic factors considered in the risk score, which could potentially lead to misclassification of the risk score staging system. Finally, the median follow-up period was short (29 months) in our study, which may have concealed some minor long-term effects among different stage categories.

In summary, the risk score staging system proposed in this study could be useful for more detailed stratification of de novo stage IV breast cancer and reflect the outcome of individualized treatment. Further studies involving larger sample sizes and more extended observation periods should be conducted to confirm the prognostic effect and validity of this staging system.

## Methods

### Patients

Data for female breast cancer diagnosed between 2010 and 2014 were extracted from the population-based SEER database^37^. Patients diagnosed with de novo stage IV breast cancer were included. Patients with de novo stage IV breast cancer were defined as distant metastases known at the time of diagnosis or found during the initial staging workup prior to the first course of treatment. We excluded patients in which there was no pathological diagnosis, T0 stage, no data on T stage, N stage, tumor grade, HER2, ER, and PR status were also excluded. The patients with unknown metastatic sites, including bone, brain, liver, and lung, were also excluded. Moreover, patients without chemotherapy in HoR+/HER2+, HoR−/HER2+, and HoR−/HER2− subtypes were also excluded from this study. Our study was exempt from approval by the Institutional Review Board of the First Affiliated Hospital of Xiamen University because the SEER program provides de-identified information of patients.

### Variables

The following variables of interest were extracted: age at diagnosis, race/ethnicity, histology, T stage, N stage, histological grade, ER status, PR status, HER2 status, radiotherapy, surgical procedures, and chemotherapy. In addition, the patterns of distant metastasis, including bone, brain, liver, lung, and other sites of metastasis, were included. TNM stage was determined based on the AJCC 7th staging system.

### Statistical analysis

The primary outcome in the present study was BCSS, which was considered as the time from the initial diagnosis to death from breast cancer. The median BCSS and BCSS rate was estimated using the Kaplan–Meier method, and the effect of various subgroups on BCSS were compared by the log-rank test. ROC curve was used to evaluate the AUC, in order to compare the effect of different risk score staging systems in predicting BCSS. The independent prognostic factors associated with BCSS were determined with the multivariate Cox proportional hazard model. Sensitivity analyses focused on the T stage, N stage, the sites of distant metastasis, and the number of distant metastasis were performed. All data were analyzed by IBM SPSS version 22.0 (IBM Corp., Armonk, NY) and MedCalc 13.0 software (MedCalc Software BVBA, Ostend, Belgium). A *P* value < 0.5 was considered to indicate the statistical significance, and all tests were two-sided.

### Reporting summary

Further information on experimental design is available in the Nature Research Reporting Summary linked to this paper.

## Supplementary information

Reporting Summary Checklist FLAT

## Data Availability

The datasets supporting the findings of this study were extracted from the population-based SEER database (https://seer.cancer.gov/). The data will be made available to researchers who have obtained permission from the SEER programme. Please contact the corresponding author, Dr Juan Zhou, email address: zhoujuan@xmu.edu.cn, or the SEER program, https://seer.cancer.gov/seertrack/data/request/, for data access requests. A metadata record describing the datasets generated and analysed during the current study, is available in figshare: 10.6084/m9.figshare.12668543^38^.

## Code availability

The codes used during the current study are available from the corresponding author on reasonable request without undue qualification.
