# Supplementary material for: Incorporation of biologic factors for the staging of de novo stage IV breast cancer
Source: NPJ Breast Cancer. 2020 Sep 7;6:43. doi: 10.1038/s41523-020-00186-5 (PMC7477201; doi:10.1038/s41523-020-00186-5)
Supplement: Supplementary file 1 — Reporting Summary Checklist FLAT [file 41523_2020_186_MOESM1_ESM.pdf]

nature research

Corresponding author(s): Juan Zhou and San-Gang Wu

Last updated by author(s): Jul 13, 2020

Reporting Summary

Nature Research wishes to improve the reproducibility of the work that we publish. This form provides structure for consistency and transparency in reporting. For further information on Nature Research policies, see Authors & Referees and the Editorial Policy Checklist.

Statistics

For all statistical analyses, confirm that the following items are present in the figure legend, table legend, main text, or Methods section.

n/a	Confirmed	
☐	☒	The exact sample size (*n*) for each experimental group/condition, given as a discrete number and unit of measurement
☐	☒	A statement on whether measurements were taken from distinct samples or whether the same sample was measured repeatedly
☐	☒	The statistical test(s) used AND whether they are one- or two-sided<br>*Only common tests should be described solely by name; describe more complex techniques in the Methods section.*
☐	☒	A description of all covariates tested
☐	☒	A description of any assumptions or corrections, such as tests of normality and adjustment for multiple comparisons
☐	☒	A full description of the statistical parameters including central tendency (e.g. means) or other basic estimates (e.g. regression coefficient) AND variation (e.g. standard deviation) or associated estimates of uncertainty (e.g. confidence intervals)
☒	☐	For null hypothesis testing, the test statistic (e.g. *F*, *t*, *r*) with confidence intervals, effect sizes, degrees of freedom and *P* value noted<br>*Give P values as exact values whenever suitable.*
☒	☐	For Bayesian analysis, information on the choice of priors and Markov chain Monte Carlo settings
☒	☐	For hierarchical and complex designs, identification of the appropriate level for tests and full reporting of outcomes
☒	☐	Estimates of effect sizes (e.g. Cohen's *d*, Pearson's *r*), indicating how they were calculated

Our web collection on statistics for biologists contains articles on many of the points above.

Software and code

Policy information about availability of computer code

Data collection	Data for female breast cancer diagnosed between 2010 and 2014 were extracted from the population-based SEER database.
Data analysis	The median BCSS and BCSS rate was estimated using the Kaplan-Meier method, and the effect of various subgroups on BCSS was compared by the log-rank test. ROC curve was used to evaluate the AUC, in order to compare the effect of different bioscore staging systems in predicting BCSS. The independent prognostic factors associated with BCSS were determined with the multivariate Cox proportional hazard model. Sensitivity analyses focused on the T stage, N stage, the sites of distant metastasis, and the number of distant metastasis were performed.

For manuscripts utilizing custom algorithms or software that are central to the research but not yet described in published literature, software must be made available to editors/reviewers. We strongly encourage code deposition in a community repository (e.g. GitHub). See the Nature Research guidelines for submitting code & software for further information.

Data

Policy information about availability of data

All manuscripts must include a data availability statement. This statement should provide the following information, where applicable:

- Accession codes, unique identifiers, or web links for publicly available datasets
- A list of figures that have associated raw data
- A description of any restrictions on data availability

The data that support the findings of this study are available from the SEER program [https://seer.cancer.gov/], which only be made available with permission from SEER program.

Field-specific reporting

Please select the one below that is the best fit for your research. If you are not sure, read the appropriate sections before making your selection.

☒ Life sciences ☐ Behavioural & social sciences ☐ Ecological, evolutionary & environmental sciences

For a reference copy of the document with all sections, see nature.com/documents/nr-reporting-summary-flat.pdf

Life sciences study design

All studies must disclose on these points even when the disclosure is negative.

Sample size	Data for female breast cancer diagnosed between 2010 and 2014 were extracted from the population-based SEER database.18 Patients diagnosed with de novo stage IV breast cancer were included.
Data exclusions	We excluded patients in which there was no pathological diagnosis, T0 stage, no data on T stage, N stage, tumor grade, HER2, ER, and PR status were also excluded. The patients with unknown metastatic sites, including bone, brain, liver, and lung, were also excluded. Moreover, patients without chemotherapy in HoR+/HER2+, HoR-/HER2+, and HoR-/HER2- subtypes were also excluded from this study.
Replication	The following variables of interest were extracted: age at diagnosis, race/ethnicity, histology, T stage, N stage, histological grade, ER status, PR status, HER2 status, radiotherapy, surgical procedures, and chemotherapy. In addition, the patterns of distant metastasis, including bone, brain, liver, lung, and other sites of metastasis, were included. TNM stage was determined based on the AJCC 7th staging system.
Randomization	This is a retrospective population-based cohort study.
Blinding	This is a retrospective population-based cohort study.

Reporting for specific materials, systems and methods

We require information from authors about some types of materials, experimental systems and methods used in many studies. Here, indicate whether each material, system or method listed is relevant to your study. If you are not sure if a list item applies to your research, read the appropriate section before selecting a response.

Materials & experimental systems

n/a	Involved in the study	
☒	☐	Antibodies
☒	☐	Eukaryotic cell lines
☒	☐	Palaeontology
☒	☐	Animals and other organisms
☒	☐	Human research participants
☐	☒	Clinical data

Methods

n/a	Involved in the study	
☒	☐	ChIP-seq
☒	☐	Flow cytometry
☒	☐	MRI-based neuroimaging

Clinical data

Policy information about clinical studies

All manuscripts should comply with the ICMJE guidelines for publication of clinical research and a completed CONSORT checklist must be included with all submissions.

Clinical trial registration	This is a retrospective population-based cohort study.
Study protocol	This is a retrospective population-based cohort study.
Data collection	Data for female breast cancer diagnosed between 2010 and 2014 were extracted from the population-based SEER database.
Outcomes	The primary outcome in the present study was breast cancer-specific survival, which was considered as the time from the initial diagnosis to death from breast cancer.
